# A highly sensitive and selective spectrofluorimetric method for the determination of manganese at nanotrace levels in some real, environmental, biological, soil, food and pharmaceutical samples using 2-(α-pyridyl)-thioquinaldinamide

**DOI:** 10.1039/c7ra12762f

**Published:** 2018-02-01

**Authors:** M. Jamaluddin Ahmed, M. Tazul Islam, Faisal Hossain

**Affiliations:** Laboratory of Analytical Chemistry, Department of Chemistry, University of Chittagong Chittagong-4331 Bangladesh pmjahmed55@gmail.com

## Abstract

A very simple, ultra-sensitive and highly selective non-extractive spectrofluorimetric method is presented for the determination of manganese at nano-trace levels using 2-(α-pyridyl)-thioquinaldinamide (PTQA). PTQA has been proposed as a new analytical reagent for the direct non-extractive spectrofluorimetric determination of manganese(vii). This novel fluorimetric reagent, PTQA becomes oxidized in a slightly acidic (0.0125–0.05 M H_2_SO_4_) solution with manganese(vii) in absolute ethanol to produce a highly fluorescent oxidized product (*λ*_ex_ = 319 nm; *λ*_em_ = 373 nm). Constant and maximum fluorescence intensities were observed over a wide range of acidity (0.0125–0.05 M H_2_SO_4_) for the period between 5 min and 24 h. Linear calibration graphs were obtained for 0.01–800 μg L^−1^ of Mn, having a detection limit of 1 ng L^−1^; the quantification limit of the reaction system was found to be 10 ng L^−1^ and the RSD was 0–2%. A large excess of over 60 cations, anions and complexing agents do not interfere in the determination. The developed method was successfully used in the determination of manganese in several standard reference materials (alloys, steels, hair and sediments) as well as in some environmental waters (potable and polluted), biological samples (human blood, urine and hair), soil samples, food samples (vegetables, fruits, tea, rice, and wheat), fertilizer samples and pharmaceutical samples (multivitamin-mineral tablets and syrup), solutions containing both manganese(ii) and manganese(vii) speciation and complex synthetic mixtures. The results of the proposed method for assessing biological, food and vegetables samples were comparable with AAS and ICP-MS and were found to be in excellent agreement.

## Abstract 2

A very simple, ultra-sensitive and highly selective non-extractive spectrofluorimetric method is presented for the determination of manganese at nano-trace levels using 2-(α-pyridyl)-thioquinaldinamide (PTQA).

## Graphical abstract

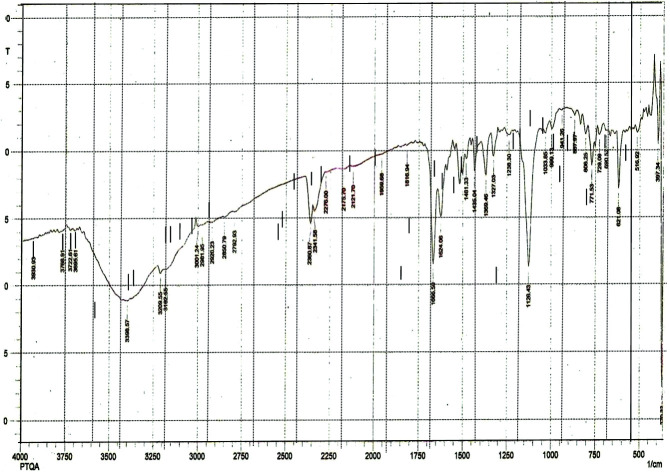

## Introduction

Manganese is an essential trace element in the metabolism of all living organisms, including plants and bacteria, and is found in all tissues of man. But manganese is also a potential carcinogen.^1^ Among the inorganic chemicals, manganese, chromium, and selenium and certain organometallic derivatives of these metals have been found to be carcinogenic under special conditions and are also considered potential inorganic genotoxic agents.^2^ The hazards of manganese have been known for a long time. The accurate determination of manganese in environmental and biological materials is of considerable importance for both metabolic and toxicological studies in humans and animals because of its dose-dependent harmful and beneficial roles. Until recently, analytical techniques of satisfactory sensitivity and accuracy have not been widely available and attempts by many researchers to use unsatisfactory procedures have led to the publication of much data and also to biochemical conclusions of dubious value.^3^ Clearly, major problems which have given rise to this situation are the very low concentrations of manganese in biological samples, and a lack of awareness of the need to exercise control over the extraneous contamination and interfering of foreign ions during all the steps of an analytical procedure. The present method that is being recommended over the existing methods almost in every respect in their own terms-selectivity, range of determination, accuracy, sensitivity, simplicity and rapidity of the operation, stability of the fluorescent system and acidity of wide variation, *etc.*

In expanding analytical fields such as environmental, biological and material monitoring of trace metals, there is an increasing need to develop the simple, sensitive and selective analytical techniques that do not use expensive or complicated test equipment. Many sophisticated technique, such as pulse polarography, NAA, HPLC, spectrophotometry, spectrofluorimetry, AAS, FAAS, ICP-OES and ICP-MS have been widely applied to the determination of manganese. However, the spectrofluorimetric method still has the advantages of being simple and without requiring expensive or complicated test equipment. For this reason, a wide variety of spectrofluorimetric methods for determination of manganese has been developed. Several authors have reported on the extractive spectrofluorimetric determination of manganese using complexes formed variety of reagents.^4–40^ In most of the methods^4–40^ cited above, manganese forms soluble or insoluble complexes with reagents with various organic solvents for spectrofluorimetric determination. Most of these reagents are expensive and non-recoverable. Most of the organic solvents which were used are carcinogenic.

The aim of this study was to develop a simpler direct spectrofluorimetric method for the nano-trace determination of manganese. In the search for a more sensitive reagent, in this work a new reagent was synthesized according to the method of Porter^41^ and a oxidation reaction of 2-(α-pyridyl)-thioquinaldinamide (PTQA); with Mn(vii) and forms an intensely fluorescent oxidized product. The method possesses distinct advantages over existing methods^4–40^ with respect to sensitivity, selectivity, range of determination, simplicity, speed, pH/acidity range, thermal stability, accuracy, precision and ease of operation. The method is based on the oxidative reaction of non-fluorescent PTQA in a slightly acidic (0.0125–0.05 M H_2_SO_4_) solution with Mn(vii) in presence of ethanol to produce a highly fluorescent oxidized product, followed by a direct measurement of the fluorescence intensity in an aqueous solution at room temperature. Oxidation is very rapid and no extraction is required. With suitable masking, the reaction can be made to be highly selective and the reagent blank solutions do not show any fluorescence.

## Experimental section

### Apparatus

A Shimadzu (Kyoto, Japan) (Model-RF-5301PC) Spectrofluorophotometer and a Jenway (England, UK) (Model-3010) pH meter with combination of electrodes were used for measurements of the fluorescence intensity and pH, respectively. The calibration and linearity of the instrument were frequently checked with standard quinine sulphate (10 μg L^−1^). A Thermo Fisher Scientific (Model-iCE 3000, origin USA) atomic absorption spectrophotometer equipped with a microcomputer-controlled nitrous oxide-acetylene flame and an Inductively Coupled Plasma-Mass Spectrometer (ICP-MS), Model – ELAN DRC-II, Perkin Elmer (Toronto, Canada) were used to compare of the results. The Elemental Analyzer (Exeter Analytical Inc. Model: CE 440) equipped with supersensitive thermal conductivity detector for simultaneous determination of CHN was used. Infrared spectrum was recorded with FTIR Spectrophotometer, Shimadzu (Model-IR Prestige 21, Detector-DTGS KBr) in the range 7500–350 cm^−1^ and model: JEOL 500SS, magnetic field strength: 500 MHz, solvent used: DMSO D6, standard: TMS, four channel NMR spectrometer with signal-to-noise ratio of ∼5000 : 1 for proton were used for characterization of the ligand.

### Synthesis and characterization of the reagent

#### Synthesis of the reagent (PTQA)

**Scheme 1:**
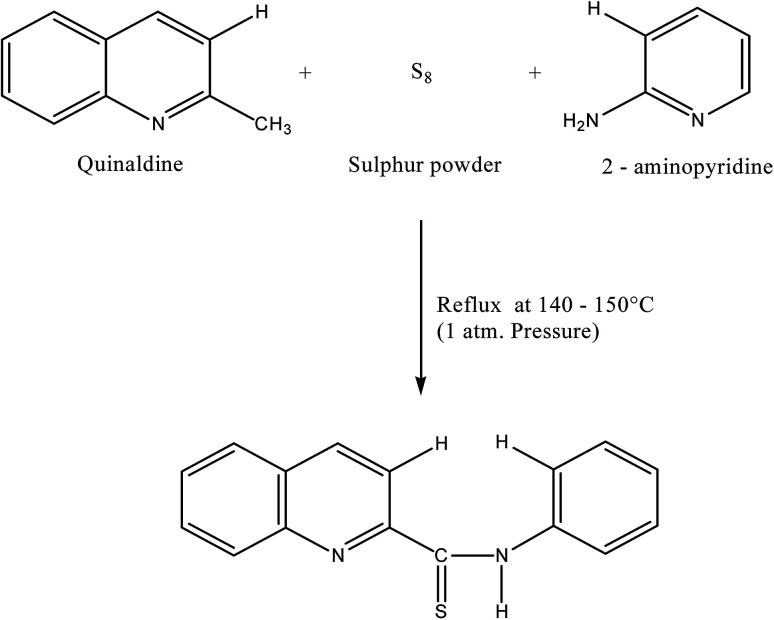
Reaction scheme of 2-(α-pyridyl)-thioquinaldinamide (PTQA).

2-(α-Pyridyl) thioquinaldinamide (PTQA, C_15_H_11_N_3_S). (molecular wt. = 265.18) was synthesized according to the method of Porter.^41^ The mixture containing 2-aminopyridin, quinaldine and sulphur powder in the molar ratio of 2 : 1 : 1.5 were mixed and refluxed for 6 hours in 250 mL round bottom flask fitted with bulb condenser under controlled temperature (140–150) °C at 1 atm. Pressure over oil bath. The reaction mixture was kept overnight. The thiocompound was filtered and crystallized using petroleum ether (60–80)° C to give a bright yellow crystalline (needle shaped) solid. The compound recrystallized from lime-distilled ethanol and was kept under vacuum (0.1 mm of Hg) for 24 hours. Yield of the product was 70%. The structure of the reagent is shown in Scheme 1.

#### Characterization of the reagent

The reagent (PTQA) was characterized by taking the melting point, elemental analysis and an FTIR spectrum and ^1^HNMR spectrum and thermogravimetric analysis. The melting point of the synthesized compound (PTQA) was 155 ± 2 °C (lit. 155 ± 1 °C)^41^ which indicated the purity of PTQA.

**Fig. 1:**
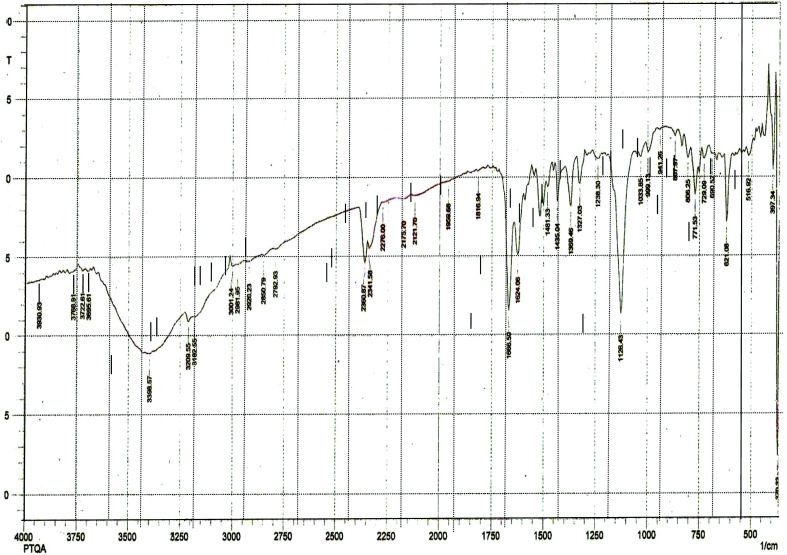
FTIR spectrum of 2-(α-pyridyl) thioquinaldinamide.

**Fig. 2:**
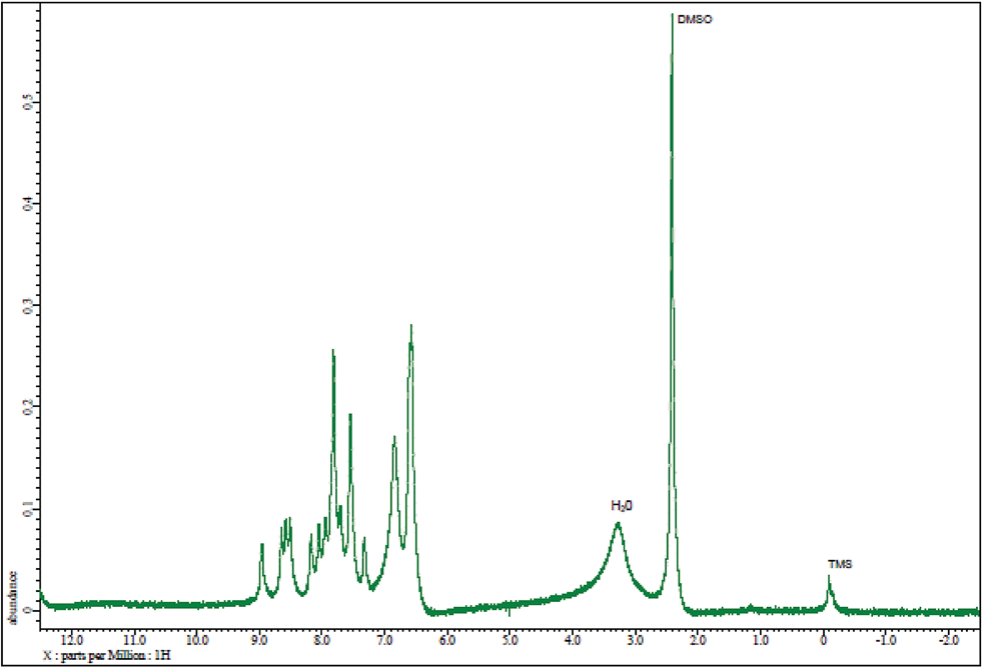
^1^HNMR spectrum of 2-(α-pyridyl)-thioquinaldinamide.

The results elemental analysis (C = 72.25, N = 13.35 and H = 4.25%) of the reagent are very in good agreement with the calculated values (C = 72.43, N = 13.55 and H = 4.55%). The FTIR spectrum of prepared reagent (PTQA) is shown in Fig. 1. The presence of FTIR peak at 1126.43 cm^−1^ in Fig. 1 was due to the characteristic C=S double bond peak (ν^C=S^, 1050–1200 cm^−1^)^41^ of the reagent indicating the formation of PTQA. Both FTIR spectral and elemental analysis data indicated the formation of the reagent PTQA. The formation of the reagent also tested by ^1^HNMR spectrum is shown in Fig. 2. The steadiness of the thermogravimetric curve obtained for about 1 g of the reagent at 80–90 °C indicated that the reagent did not contain any moisture.

The elemental analysis were performed by the National Center of Excellence in Analytical Chemistry, University of Sindh, Pakistan and FTIR spectra was recorded with FTIR spectrophotometer, Shimadzu (Model-IR Prestige 21, Detector-DTGS KBR) in the range 7500–350 cm^−1^ from our laboratory and ^1^HNMR spectrum was recorded with ^1^HNMR spectrophotometer model: JEOL 500SS from Kanazawa University, Japan.

#### Live subject statement

We were not aiming to carry out detailed human studies but some samples from individuals were used in our study and as such we abided by all the necessary procedures and regulations and our University gave consent. University of Chittagong, Bangladesh is committed to the protection and safety of human subjects involved in research.

### Reagents and solutions

All the chemicals used were of analytical reagent grade of the highest purity available. High-purity absolute ethanol and high-purity de-ionized water were used throughout. High-purity water was obtained by passing tap water through cellulose absorbent and to mixed-bed ion exchange columns, followed by distillation in a corning AG-11 unit. Glass vessel were cleaned by soaking in acidified solutions of KMnO_4_ or K_2_Cr_2_O_7_ followed by washing with concentrated HNO_3_ and rinsed several times with high purity de-ionized water. Stock solutions and environmental water sample (1000 mL each) were kept in polypropylene bottles containing 1 mL concentrated HNO_3_. More rigorous contamination control was used when the manganese levels in the specimens were low.

### PTQA solution (1 × 10^−3^ M)

The reagent solution was prepared by dissolving the requisite amount (0.0024 g) of PTQA, in a known volume (10 mL) of absolute ethanol. A freshly prepared reagent solution (10^−4^ M) was used whenever required.

### Manganese(vii) standard solution (1.82 × 10^−2^ M)

A 100 mL amount of stock solution (1 mg mL^−1^) of heptavalent manganese was prepared by dissolving 287.66 mg of potassium permanganate (KMnO_4_) (Aldrich A.C.S. grade) in doubly distilled de-ionized water. Aliquots of this solution were standardized with oxalic acid.^42^ More dilute standard solutions were prepared by appropriate dilution of aliquots from the stock solution with de-ionized water when required. A freshly standardized solution was always used.

### Mn(ii) standard solution (1.82 × 10^−2^ M)

A 100 mL amount of stock solution (1 mg L^−1^) of divalent manganese was prepared by dissolving 307.7 mg of purified-grade (E Merck proanalysis grade) monohydrated manganese sulphate (MnSO_4_·H_2_O) (super special grade J. T. Baker) in doubly distilled de-ionized water. Aliquots of this solution were standardized titrimetrically with disodiumdihydrogenethylenediaminetetraacetate (Na_2_H_2_EDTA) using Eriochrome Black T.^42^ More dilute standard solutions were prepared by appropriate dilution of aliquots from the stock solution with de-ionized water as and when required.

### Potassium dichromate solution

A 100 mL amount of stock solution (0.1 N) was prepared by dissolving 500 mg of finely powdered K_2_Cr_2_O_7_ (Merck) in 100 mL de-ionized water.

### Ammonium persuphate solution

Ammonium persuphate solution (2% w/v) (A.C.S.-grade 99% pure) was freshly prepared by dissolving 2 g in 100 mL of de-ionized water.

### Tartrate solution

A 100 mL stock solution of tartrate (0.01% w/v) was prepared by dissolving 10 mg of A.C.S.-grade (99%) potassium sodium tartrate tetrahydrate in (100 mL) de-ionized water.

### Aqueous ammonia solution

A 100 mL solution of an aqueous ammonia solution was prepared by diluting 10 mL concentrated NH_4_OH (28–30%, A.C.S.-grade) to 100 mL with de-ionized water. The solution was stored in a polypropylene bottle.

### EDTA solution

A 100 mL stock solution of EDTA (0.01% w/v) was prepared by dissolving 10 mg A.C.S.-grade (≥99%) ethylenediaminetetraacetic acid as disodium salt dihydrate in (100 mL) de-ionized water.

### Other solutions

Solutions of a large number of inorganic ions and complexing agents were prepared from their AnalaR grade or equivalent grade water-soluble salts (or the oxides and carbonates in hydrochloric acid); those of niobium, tantalum, titanium, zirconium and hafnium were specially prepared from their corresponding oxides (Specpure, Johnson Matthey) according to the recommended procedures of Mukharjee.^43^In the case of insoluble substances, special dissolution methods were adopted.^44^

### Procedure

To 0.1–1.0 mL of a neutral aqueous solution containing 0.1–8000 ng of manganese(vii) in a 10 mL calibrated flask was mixed with a 1 : 70–1 : 300 fold molar excess (preferably 1 mL of 1 × 10^−4^ M) of the 2-(α-pyridyl) thioquinaldinamide (PTQA) reagent solution followed by the addition of 0.5–2 mL (preferably 1 mL) of 0.025 M of sulfuric acid. The solution was mixed well and allowed to stand for 5 min after which 2 mL of absolute ethanol was added and the mixture was diluted to the mark with de-ionized water. The fluorescence intensity of the system was measured at 373 nm against a corresponding reagent blank, prepared concurrently, keeping the excitation wavelength maximum at 319 nm and the instrument setting the same. The manganese content in an unknown sample was determined using a concurrently prepared calibration graph.

### Sample collection and preservation

#### Environmental samples

Water and soil samples were collected in polythene bottles from different places of Bangladesh. After collection, HNO_3_ (1 mL L^−1^) was added as preservative.

#### Blood and urine

Blood and urine samples were collected in polythene bottles from effected persons of Chittagong Medical College Hospital, Bangladesh. Immediately after collection they were stored in a salt–ice mixture and latter, at the laboratory, were at −20 °C.

#### Soil samples

Soil samples were collected from different locations of Bangladesh. Samples were dried in air and homogenized with a mortar.

#### Food samples

Food samples (rice, wheat, tea, fruits and vegetables) were collected from local market of Chittagong. After collection the samples (fruits and vegetables) were stored in refrigerator for preservation. Samples (rice, wheat, tea) were used as dry condition and homogenized with a mortar.

#### Pharmaceutical samples

Pharmaceutical samples (tablet and syrup) of different companies were collected from local pharmacy of Chittagong. Samples (tablet) were homogenized with a mortar.

#### Fertilizer samples

Fertilizer samples of different verities were collected from local market of Chittagong. Samples were homogenized with a mortar.

## Results and discussion

### Factors affecting the fluorescence intensity

#### Spectral characteristics

**Fig. 3:**
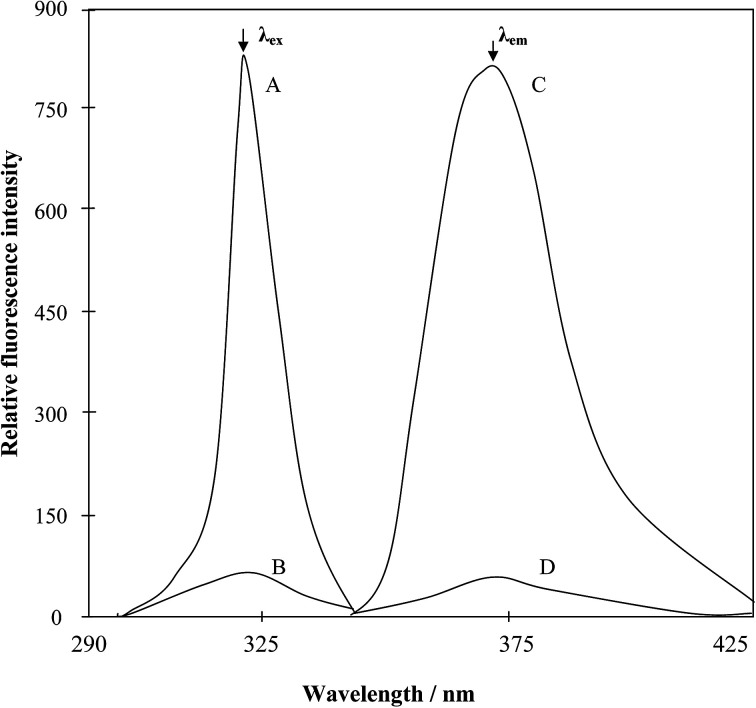
(A and B), Excitation spectra of the Mn^VII^–PTQA system and the reagent blank (*λ*_em_ = 373 nm); and (C and D) are the corresponding emission spectra (*λ*_ex_ = 319 nm).

The excitation and emission spectra of the fluorescent Mn(vii)–PTQA in 0.025 M sulfuric acid medium was recorded using the spectrofluorophotometer. The excitation and emission maxima were at 319 nm and 373 nm, respectively. The reagent blank exhibited negligible fluorescence, despite having wavelength maximum in the same region. In all instances, measurements were made against the reagent blank. The spectra are shown in Fig. 3.

### Optimization of some parameters on the fluorescence intensity

#### Effect of solvent

**Fig. 4:**
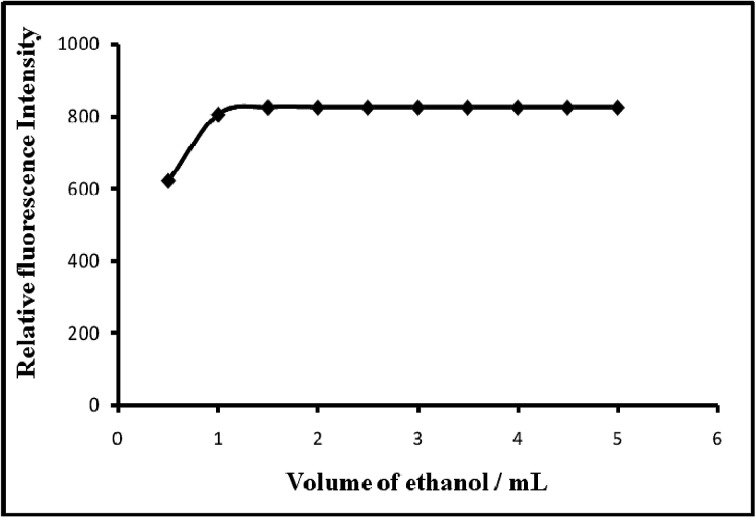
Effect of solvent (ethanol) on the fluorescence of Mn(vii)–PTQA system.

Because PTQA is insoluble in water, an organic solvent was used for the system. Of the various solvents [chloroform, benzene, carbon tetrachloride, *n*-butanol, isobutanol, ethanol, 1,4-dioxane and *N*,*N*-dimethylformamide (DMF)] were tested for the system, ethanol was found to be the best solvent for the system. The effect of ethanol on the fluorescence intensity was studied and no adverse effect was observed over a wide range of ethanol concentrations. It was observed that Mn(vii)–PTQA system with 10 μg L^−1^ of Mn in absolute ethanol solution produced a constant fluorescence intensity as shown in Fig. 4. A concentration of 20% v/v ethanol in the final volume was sufficient to prevent any precipitation or turbidity and to allow accurate measurements. Therefore, a 20% v/v ethanolic solution was used in the recommended procedure.

#### Effect of acidity

**Fig. 5:**
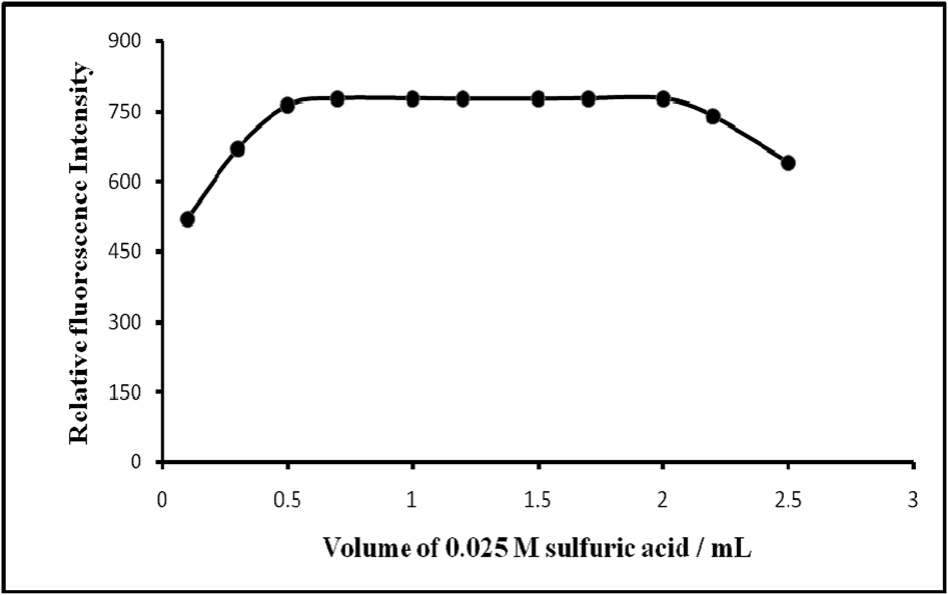
Effect of acidity on the fluorescence of Mn(vii)–PTQA system.

Of the various acids (nitric, sulfuric, hydrochloric and phosphoric) studied, sulfuric acid was found to be the best acid for the system. Although H_3_PO_4_ was used previously to be most suitable acid^45^ for the oxidation of manganese(ii) but the proposed procedure for the spectrofluorimetric determination of manganese(vii) with PTQA H_2_SO_4_ is more better than H_3_PO_4_. The fluorescence intensity was at maximum and constant when the 10 mL of solution (10 μg L^−1^ of (VII)) contained 0.5–2.0 mL of 0.025 M sulfuric acid at room temperature (25 ± 5 °C). Outside this range of acidity, the fluorescence intensity decreased (Fig. 5). The optimum acidity range in the final solution is therefore 0.0125–0.05 M H_2_SO_4_. For all subsequent measurements 1 mL of 0.025 M sulfuric acid was added.

#### Effect of temperature

The Mn(vii)–PTQA system attained maximum and constant fluorescence intensity at (15–40) °C temperature. For all subsequent measurements was done at room temperature (25 ± 5 °C).

#### Effect of time

The reaction is instantaneous. The Mn(vii)–PTQA system attained maximum and constant fluorescence intensity immediately (within 5 min) after dilution of the solution to the final volume, which then remained strictly unaltered for 24 h at room temperature (25 ± 5 °C).

#### Effect of reagent concentration

**Fig. 6:**
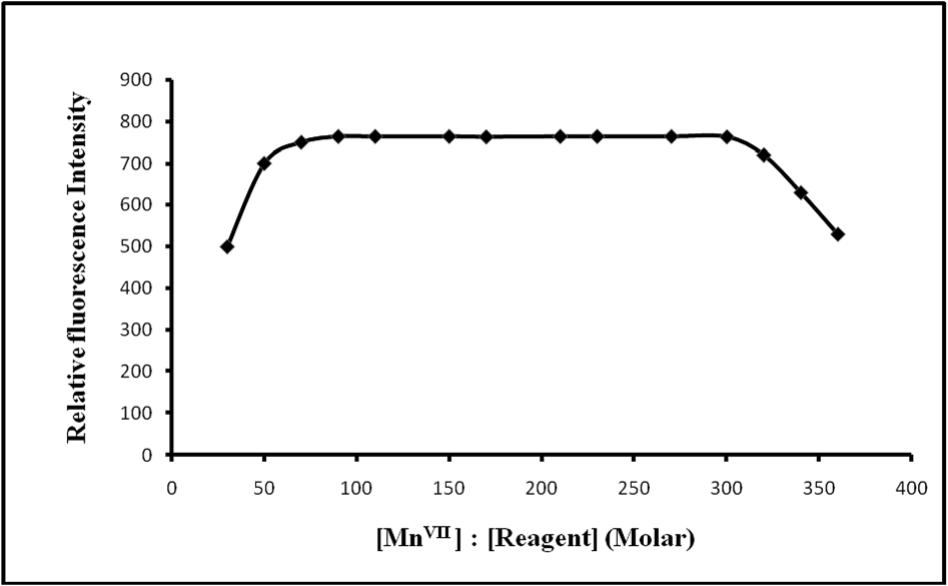
Effect of reagent on the fluorescence of Mn(vii)–PTQA system.

Different molar excesses of PTQA were added to a fixed metal ion concentration and fluorescence intensities were measured according to the standard procedure. It was observed that at 10 μg L^−1^ Mn(vii) metal and the reagent molar ratios of 1 : 70–1 : 300 produced a constant fluorescence intensity of the oxidized product. Outside this range of reagent, the fluorescence intensity decreased (Fig. 6). At different manganese(vii) concentrations (0.5 and 1 μg L^−1^), the effect of varying the reagent concentration was similar. For all subsequent measurements 1 mL of 1 × 10^−4^ M PTQA reagent was added.

#### Effect of metal concentration

The well-known equation for spectrofluorimetric analysis in very dilute solutions derived from Beer's law. The effect of metal concentration was studied over 0.001–1000 μg L^−1^ distributed in six different sets (0.001–0.01, 0.01–0.1, 0.1–1, 1–10, 10–100 and 100–1000 μg L^−1^) for convenience of measurement. The fluorescence intensity was linear over a wide range [10 pg mL^−1^ to 800 ng mL^−1^ for 0.01–800 μg L^−1^] of manganese at excitation wavelength at 319 nm and emission wavelength at 373 nm representing five linear graphs (0.01–0.1, 0.1–1.0, 1–10, 10–100 and 100–1000 μg L^−1^). Of five calibration graphs, the one showing the limit of the linearity range (Fig. 7); the next four were straight-line graphs passing through the origin (*R*^2^ = 0.9998). The limit of detection and limit of quantization were found to be 1 pg mL^−1^ and 10 pg mL^−1^, respectively. The selected analytical parameters obtained with the optimization experiments are summarized in Table 1.

**Fig. 7:**
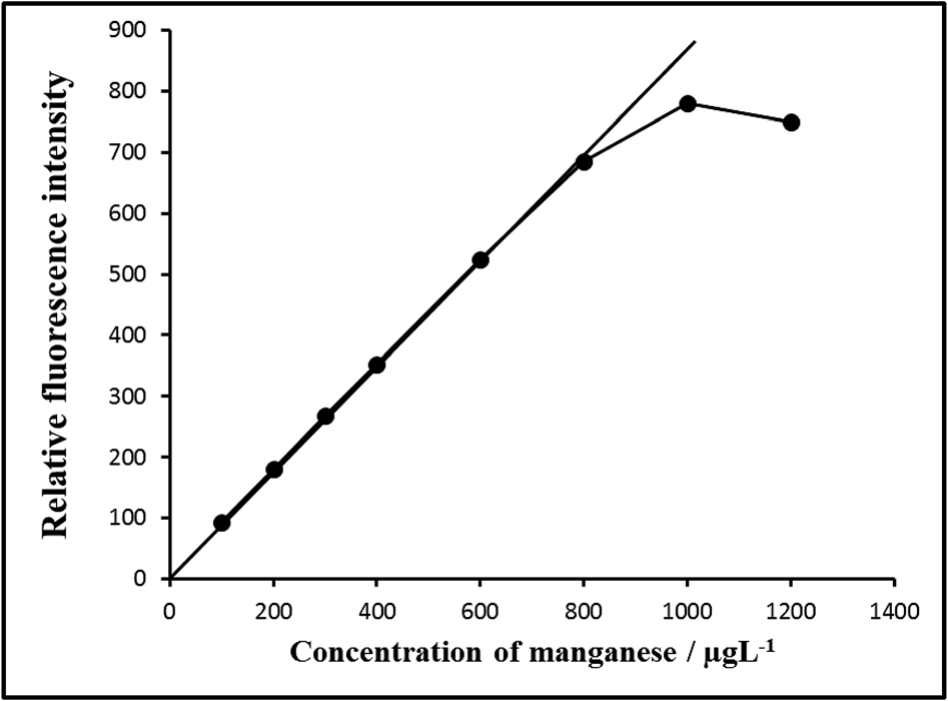
Calibration graph E: 100–800 μg L^−1^ of manganese(vii). Bandwidth: Ex. slit-1.5, Em. slit-1.5, sensitivity: high.

**Table:** Selected analytical parameters obtained with the optimization experiments

Parameters	Studied range	Selected value
Excitation wavelength maximum/*λ*_ex_ (nm)	200–700	319
Emission wavelength maximum/*λ*_em_ (nm)	200–700	373
Solvent/amount of absolute ethanol/mL	0–6	1–5 (preferably 2)
Acidity/M H_2_SO_4_	0.001–0.075	0.0125–0.05 (preferably 0.025)
pH	3.69–0.69	3.0–1.0 (preferably 1.3)
Time/h	0–72	1 min–24 h (preferably 5 min)
Temperature/°C	10–70	15–40 (preferably 25 ± 5)
Reagent (fold molar excess, M : R)	1 : 1–1 : 500	1 : 70–1 : 300 (preferably 1 : 100)
Linear range/μg L^−1^	0.001–1000	0.01–800
Limit of quantification/ng L^−1^	1–100	10
Detection limit/ng L^−1^	0.01–10	1
Reproducibility (% RSD)	0–10	0–2%
Regression co-efficient (*R*^2^)	0.9992–0.9999	0.9998

#### Effect of foreign ions

More than 60 anions, cations and complexing agents were studied individually to investigate their effect on the determination of 10 μg L^−1^ of manganese(vii). The criterion for an interference^45^ was a fluorescence intensity value varying by more than ±5% from the expected value for manganese alone. The results are summarized in Table 2. As can be seen a large number of ions have no significant effect on the determination of manganese. The most serious interference were from Se(iv), Cr(vi) and Ce(iv) ions. Interference from these ions is probably due to oxidative product formation with PTQA.

**Table:** Table of tolerance limits of foreign ions^a^, tolerance ratio [species(x)/*M*_n_ (w/w)]

Species x	Tolerance ratio x/*M*_n_ (w/w)	Species x	Tolerance ratio x/*M*_n_ (w/w)
Ammonium(i)	1000	Lithium	1000
Arsenic(iii)	500^b^	Lead(ii)	200
Arsenic(v)	500	Magnesium	1000
Aluminum	200	Manganese(ii), manganese(iv)	1000, 500
Azide	200	Mercury(ii)	200
Ascorbic acid	1000	Molybdenum(vi)	500
Antimony	500	Nitrate	1000
Bromide	100	Nickel	500
Bismuth(iii)	500	Oxalate	1000
Beryllium(ii)	1000	Potassium	1000
Calcium	1000	Phosphate	1000
Chloride	1000	Selenium(vi)	500
Cobalt(ii)	200^c^	Selenium(iv)	50^c^
Cobalt(iii)	500	Silver	500
Chromium(iii)	1000	Sodium	1000
Chromium(vi)	100^c^	Strontium	500
Cadmium	1000	Sulfate	500
Carbonate	1000	Titanium(iv)	100
Cesium	1000	Tellurium(iv)	500
Citrate	1000	Tartrate	1000
Cerium(iii)	500	Copper(ii)	500
Cerium(iv)	500^b^	Thiocyanate	1000
Cyanide	200	Thiourea	100
EDTA	1000	Tungsten(vi)	1000
Fluoride	1000	Tin(ii)	50^b^
Iodide	1000	Tin(iv)	50^b^
Iron(ii)	200	Vanadium(v)	1000
Iron(iii)	200	Zinc	1000

a Tolerance limit was defined as ratio that causes less than ±5 percent interference.

b With 10 mg L^−1^ tartrate.

c With 10 mg L^−1^ EDTA.

The greater tolerance limits for these ions can be achieved by using several masking agents. In order to eliminate the interference of Se(iv), Cr(vi) and Ce(iv) ions, EDTA and tartrate can be used as masking agents, respectively.^46^ A 50, 100 and 500-fold excess of Se(iv), Cr(vi) and Ce(iv) ions could be masked with EDTA and tartrate, respectively. During the interference studies, if a precipitate was formed, it was removed by centrifugation. The amount mentioned is not the tolerance limit but the actual amount studied. However, for those ions whose tolerance limits have been studied, their tolerance ratios are mentioned in Table 2.

Strong reducing agents such as tin(ii), chloride, iron(ii), sulphate, hydroxylamine, hydrochloride and sodium azide, which would otherwise reduce manganese(vii), undergo oxidation during the treatment of manganese(ii) solution with persulphate and hence are not a problem.

#### Precision and accuracy

The precision of the present method was evaluated by determining different concentrations of manganese (each analyzed at least five times). The relative standard deviation (*n* = 5) was 2–0% for 0.1–8000 ng of manganese(vii) in 10 mL, indicating that this method is highly precise and reproducible (Table 1). The detection limit (3 s of the blank) and limit of quantization (10 times of detection limit) for manganese(vii) were found to be 1 ng L^−1^ and 10 ng L^−1^, respectively. The method was also tested by analyzing several synthetic mixtures containing manganese(vii) and diverse ions. The results for total manganese were in excellent agreement with certified values (Table 3). The reliability of the procedure was tested by recovery studies. The average percentage recovery obtained for addition of manganese(vii) spike to some environmental water samples was quantitative, as shown in Table 4. The results of biological analyses by the spectrofluorimetric method were in excellent agreement with those obtained by AAS (Table 5). The results of vegetable and fruit samples analyzed by the present method were found to be very much comparable with those found by AAS (Table 8). The results of food analyses by spectrofluorimetric method were also found to be in excellent agreement with those obtained by ICP-MS (Table 9). The results of speciation of manganese(ii) and manganese(vii) in mixtures were highly reproducible (Table 10). Statistical comparison of proposed method with reference methods are shown in Table 11. Hence, the precision and accuracy of the method were found to be excellent.

**Table:** Determination of manganese in certified reference materials

Sample	Certified reference materials (composition, %)	Manganese (%)		
		In CRM sample	Found (*n* = 5)	RSD^a^
1	BCS-CRM 163, unalloyed steel (C = 1.2, Mn = 0.472, Ni = 0.608, Si = 0.18, Cu = 0.05, Cr = 0.03)	0.472	0.47	1.5
2	BAS-CRM-10g, high-speed brass (Cu = 60.8, Fe = 1.56, Pb = 0.23, Ni = 0.16, Sn = 0.21, Al = 3.34, Zn = 32.0, Mn = 1.36)	1.36	1.35	1.2
3	BCS-CRM-238, unalloyed steel (C = 0.2, Si = 0.06, S = 0.05, Mn = 0.51, Ni = 0.06, Cr = 0.06 and Cu = 0.05)	0.51	0.50	2.0
4	BAS-CRM-1d, mild steel (C = 0.19, Si = 0.06, S = 0.04, Mn = 0.52, P = 0.04)	0.52	0.518	1.8
5	BAS-CRM-33b, alloy cast iron (Mn = 0.64, Cr = 0.61, Mo = 0.04, Ni = 2.24)	0.64	0.625	1.6
6	CRM-BCR-397, human hair	11.2 ± 0.3^b^	11.15 ± 0.5	1.0
7	NIST-SRM-1646, estuarian sediment	234.5^c^	232.0	1.5

a The measure of precision is the relative standard deviation (RSD).

b Values in μg g^−1^.

c Values in mg kg^−1^.

**Table:** Determination of manganese in some environmental water samples

Sample		Manganese/μg L^−1^		Recovery ± *s* (%)	*s*_r_ b (%)
		Added	Found^a^ (*n* = 5)		
Tap water		0, 10, 50	55.0, 65.0, 108.0	100.0 ± 0.0, 103.0 ± 0.5	0.00, 0.25
Well water		0, 10, 50	35.0, 45.0, 85.0	100 ± 0.0, 100 ± 0.0	0.00, 0.00
River water	Karnaphully (upper)	0, 10, 50	75.0, 88.0, 125.0	104.0 ± 06, 100.0 ± 0.0	0.35, 0.00
	Karnaphully (lower)	0, 10, 50	80.5, 90.0, 135.0	99.5 ± 0.5, 103.0 ± 0.8	0.21, 0.39
	Halda (upper)	0, 10, 50	45.0, 55.0, 98.0	100.0 ± 0.00, 103.0 ± 0.6	0.00, 0.29
	Halda (lower)	0, 10, 50	53.0, 65.0, 105.0	103.0 ± 0.8, 102.0 ± 0.5	0.36, 0.33
Sea water	Bay of Bengal (upper)	0, 10, 50	25.0, 35.0, 80.0	100.0 ± 0.0, 106.0 ± 1.0	0.00, 0.45
	Bay of Bengal (lower)	0, 10, 50	35.0, 45.0, 90.0	100.0 ± 0.0, 105.8 ± 1.0	0.00, 0.38
Drain water	T. S. P. Complex^c^	0, 100, 500	130.0, 235.0, 630.0	102.0 ± 0.8, 100.2 ± 0.0	0.18, 0.00
	PHP^d^	0, 100, 500	160.0, 260.0, 665.0	100.0 ± 0.0, 100.7 ± 0.5	0.00, 0.25
	BSRM^e^	0, 100, 500	245.0, 350.0, 750.0	101.0 ± 0.8, 100.6 ± 1.0	0.29, 0.35
	K. P. M. water^f^	0, 100, 500	188.0, 288.0, 690.0	100.0 ± 0.8, 100.2 ± 0.5	0.00, 0.42
	Berger paints^g^	0, 100, 500	270.0, 370.0, 775.0	100.0 ± 0.0, 100.6 ± 1.2	0.00, 0.55

a Average of five replicate determinations of each sample.

b The measure precision is the relative standard deviation(*s*_r_).

c T. S. P. Complex Ltd., Patenga, Chittagong.

d PHP Glass factory, Chittagong.

e Bangladesh Steel Re-rolling Mills Ltd. (BSRM), Baizid Bosthami, Chittagong.

f Karnaphuly Paper Mills, Chandraghona, Chittagong.

g Berger Paints Bangladesh Limited, Kalurghat, Chittagong.

**Table:** Determination results of manganese for human fluids and hair

Serial no.	Sample	Manganese/μg L^−1^		Sample source^a^
		AAS (*n* = 5)	Proposed method (*n* = 5)	
1	Blood, urine	800 ± 1.0, 210.00 ± 1.5	798.5 ± 1.3, 201.5 ± 1.5	Manganism (damage of central nervous system or neurological disorder) patient (female)
2	Blood, urine	460.5 ± 1.3, 120.6 ± 1.5	465.8 ± 1.5, 121.5 ± 1.7	Leucopenia patient (female)
3	Blood, urine	385.8 ± 1.3, 106.5 ± 1.8	390.5 ± 1.5, 112.2 ± 2.0	Liver cirrhosis patient (male)
4	Blood, urine	370.6 ± 1.5, 93.5 ± 1.8	372.5 ± 1.8, 98.5 ± 2.2	Pneumonitis (manganic pneumonia) patient (male)
5	Blood, urine	230.8 ± 1.6, 85.6 ± 2.0	232.5 ± 2.0, 85.6 ± 1.8	Hypertension patient (male)
6	Blood, urine	110.0 ± 2.1, 30.5 ± 1.9	115.0 ± 2.0, 32.8 ± 2.5	Asthma patient (male)
7	Blood, urine	15.5 ± 2.0, 3.85 ± 1.5	15.8 ± 1.5, 3.82 ± 1.8	Normal adult (male)
8	Hair^b^	790.5 ± 1.8	795.8 ± 1.5	Normal human hair (male)

a Samples were from Chittagong Medical College Hospital.

b Values in ng g^−1^.

### Nature of the fluorescent species

**Fig. 8:**
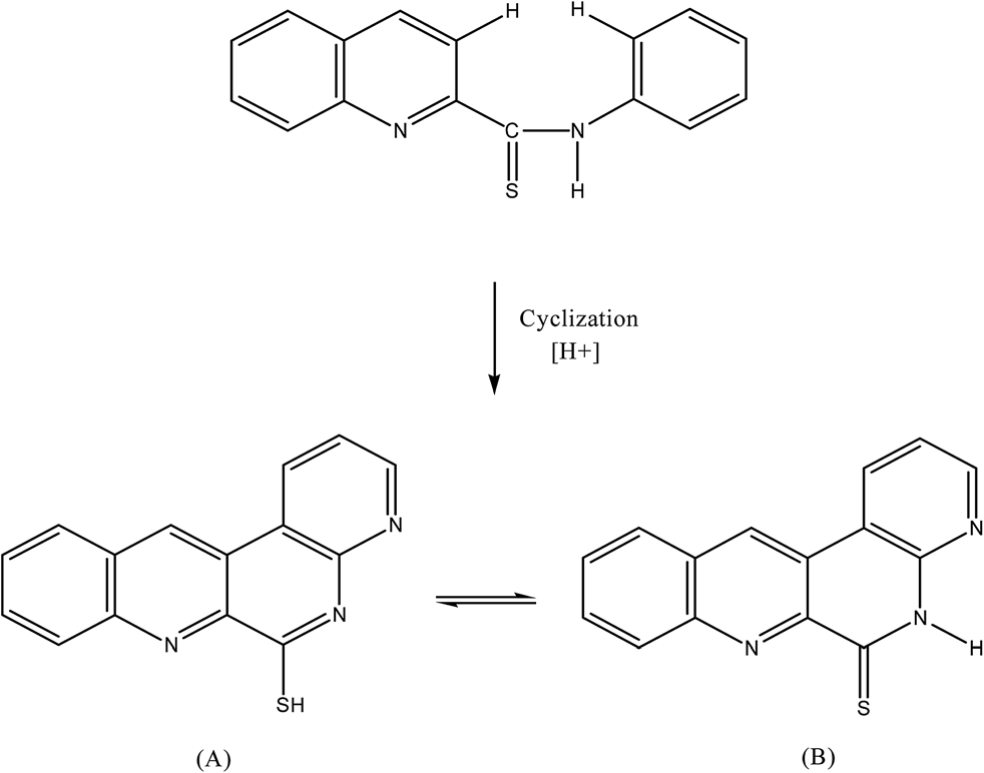
Mechanism of oxidative cyclization reaction of 2-(α-pyridyl)-thioquinaldinamide (PTQA).

The non fluorescent reagent, PTQA, produced the same spectral characteristics with excitation and emission wavelengths almost invariably around 319 nm and 373 nm, with manganese(vii), chromium(vi), selenium(iv) and cerium(iv) and with persulphate, hydrogen peroxide, and triiodide in acidic media. This indicates that the fluorescence species is an oxidized product of the reagent itself and not a chelate. Similar oxidative fluorescent reactions have been utilized previously.^47^ The PTQA reagent produced here has so many potential reaction sites that the structure of the oxidized fluorescent species is difficult to predict. Given that ring closure can lead to intense fluoresce in some circumstances, it seems likely that photo-oxidative cyclization takes place, leading to the formation of structure (A) in resonance with structure (B) (Fig. 8).

### Applications

The present method was successfully applied to the determination of manganese(vii) in a series of synthetic mixtures of various compositions and also in a number of real samples *e.g.* several Certified Reference Materials (CRM) (Table 3). The method was also extended to the determination of manganese in a number of environmental, biological, soil, food, tea, fertilizer, vegetable, fruit and pharmaceutical samples. In view of the unknown composition of environmental water samples, the same equivalent portions of each such sample were analyzed for manganese content; the recoveries in both the “spiked” (added to the samples before the mineralization or dissolution) and the “unspiked” samples are in excellent agreement (Table 4). The results of biological analyses by spectrofluorimetric method were found to be in excellent agreement with those obtained by AAS (Table 5). The results of soil analyses by the spectrofluorimetric method are shown in Table 6. The results of pharmaceutical samples by the spectrofluorimetric method are shown in Table 7. The results of vegetable and fruit samples by the spectrofluorimetric method were found to be in excellent agreement with that of found by AAS (Table 8). The results of food analyses by spectrofluorimetric method were also found to be in good agreement with those obtained by ICP-MS (Table 9). The results of speciation of manganese(ii) and manganese(vii) in mixtures are shown in Table 10. Statistical comparison of proposed method with reference methods are shown in Table 11.

**Table:** Determination of manganese in some surface soil

Serial no.	Manganese^a^ (mg kg^−1^) (*n* = 5)	RSD (%)	Sample source^b^
S_1_^b^	3.5 ± 0.5	1.8	Agriculture soil (Chittagong University Campus)
S_2_	1.05 ± 0.3	1.5	Marine soil (Bay of Bengal)
S_3_	21.5 ± 1.0	1.8	Traffic soil (Kadamtali Bus Terminal)
S_4_	95.6 ± 1.5	2.2	Industrial soil (T. S. P. Complex, Chittagong)
S_5_	78.5 ± 1.0	2.3	Industrial soil (Eastern refinery, Chittagong, Bangladesh)
S_6_	130.5 ± 1.3	2.5	Industrial soil (Eastern Cables)
S_7_	145.0 ± 1.0	2.0	Industrial soil (P·H·P glass)
S_8_	7.85 ± 0.8	2.1	Road side soil (Dhaka-Chittagong Highway)
S_9_	117.0 ± 1.5	2.0	Paint soil (Berger paint, Chittagong)
S_10_	7.5 ± 0.8	1.8	River soil (River Karnaphuly, Chittagong)

a Average of five analyses of each sample.

b Composition of the soil samples: C, N, P, K, Na, Ca, Mg, Cu, Mo, Fe, Pb, V, Zn, Mn, Co, NO_3_, SO_4_*etc.*

**Table:** Determination of manganese in some pharmaceutical samples

Pharmaceutical samples	Brand name	Trade name	Manganese/μg g^−1^ or mg L^−1^		RSD (%)
			Reported (claimed) value	Found (*n* = 5)	
Tablet	Square Pharmaceuticals Ltd.	Mulvit plus (multivitamin-mineral)/μg g^−1^	101.5	102.5	2.0
	Eskayef Bangladesh Ltd (SK + F)	Ostocal M (calcium, vitamin D & minerals)/μg g^−1^	185.5	184.2	1.5
	Beximco Pharmaceuticals Ltd (BPL)	Bextram gold (A to Z) (multivitamin-mineral)/μg g^−1^	201.5	205.0	2.0
Syrup	The ACME Laboratory	Menuvit syrup (multivitamin-mineral)/mg L^−1^	155.0	153	2.3
	Finecure Pharmaceuticals Ltd.	Menuvit syrup (multivitamin-mineral)/mg L^−1^	160.0	158.8	1.8

**Table:** Determination of manganese in some vegetable and fruit samples

Serial no.	Sample	Manganese/mg kg^−1^ Found^a^ ± *s* (*n* = 5)				Sample source
		AAS (*n* = 5)		Proposed method		
		Found	RSD^b^	Found	RSD^b^	
1	White cabbage (*Brassica oleraceacupitata*)	20.5	1.0	21.8	1.2	Local market, Chittagong
2	Radish (*Raphanus sativus*)	29.5	1.5	30.0	2.0	Local market, Chittagong
3	Tomato (*Lycopersicon esculentum*)	15.75	1.8	17.5	2.5	Local market, Chittagong
4	Cauliflower (*Brassica oleracea*)	45.5	1.6	46.5	1.5	Local market, Chittagong
5	Onion (*Allium cepa*)	75.5	1.8	78.0	2.0	Local market, Chittagong
6	Coriander seeds (*Coriandrum sativum*)	40.5	2.0	41.5	1.8	Local market, Chittagong
7	Potato (*Solanum tuberosum*)	7.50	1.4	8.50	1.5	Local market, Chittagong
8	Garlic (*Allium sativum*)	49.5	1.5	50.4	1.9	Local market, Chittagong

a Average of five replicate analyses of each sample.

b The measure of precision is the relative standard deviation (RSD).

**Table:** Determination of manganese in some food and tea samples

Sample^a^		Manganese/μg g^−1^			
		ICP-MS (*n* = 5)		Proposed method (*n* = 5)	
		Found	RSD^b^ (%)	Found	RSD^b^ (%)
Tea (*Camellia sinensis*)	Proyash tea	285.8	1.8	289.5	1.5
	Ispahani tea	282.4	1.9	285.0	1.7
	Taza tea	188.8	2.0	185.5	1.8
	Seylon tea	297.6	2.1	299.3	1.5
	Green tea	295.5	2.0	198.2	1.8
Rice (*Oryza sativa*)		1.95	1.0	1.98	1.2
Wheat (*Triticum aestivum*)		2.88	1.6	2.91	1.6

a Samples were from Local market, Chittagong.

b The measure of precision is the relative standard deviation (RSD).

Determination of manganese(ii) and manganese(vii) in mixtures

**Table:** 

Serial no.	Mn(vii) : Mn(ii)	Mn, taken (μg L^−1^)		Mn, found (μg L^−1^)		Error (μg L^−1^)	
		Mn(vii)	Mn(ii)	Mn(vii)	Mn(ii)	Mn(vii)	Mn(ii)
1	1 : 1	10	10	9.98	9.99	0.02	0.01
1	1 : 1	10	10	10.00	10.02	0.00	0.02
1	1 : 1	10	10	9.97	9.98	0.03	0.02
Mean error: Mn(vii) = ±0.017; Mn(ii) = ±0.017							
Standard deviation: Mn(vii) = ±0.012; Mn(ii) = ±0.011

**Table:** 

Serial no.	Mn(vii) : Mn(ii)	Mn, taken (μg L^−1^)		Mn, found (μg L^−1^)		Error (μg L^−1^)	
		Mn(vii)	Mn(ii)	Mn(vii)	Mn(ii)	Mn(vii)	Mn(ii)
1	1 : 5	10	50	9.98	49.80	0.02	0.20
1	1 : 5	10	50	9.99	49.70	0.01	0.30
1	1 : 5	10	50	9.98	49.80	0.02	0.20
Mean error: Mn(vii) = ±0.017; Mn(ii) = ±0.234							
Standard deviation: Mn(vii) = ±0.0015; Mn(ii) = ±0.0018

**Table:** 

Serial no.	Mn(vii) : Mn(ii)	Mn, taken (μg L^−1^)		Mn, found (μg L^−1^)		Error (μg L^−1^)	
		Mn(vii)	Mn(ii)	Mn(vii)	Mn(ii)	Mn(vii)	Mn(ii)
1	1 : 10	10	100	9.99	99.8	0.01	0.2
1	1 : 10	10	100	9.98	99.9	0.02	0.1
1	1 : 10	10	100	9.97	99.8	0.03	0.2
Mean error: Mn(vii) = ±0.02; Mn(ii) = ±0.17							
Standard deviation: Mn(vii) = ±0.0019; Mn(ii) = ±0.0018							

**Table:** Statistical comparison of proposed method with reference methods

Samples	F – test results^a^				
	(*s*_1_^2^/*s*_2_^2^)^15^	(*s*_1_^2^/*s*_3_^2^)^26^	(*s*_1_^2^/*s*_4_^2^)^31^	(*s*_1_^2^/*s*_5_^2^)^33^	(*s*_1_^2^/*s*_6_^2^)^28^
Water	0.127		0.58	0.23	0.0019
Water	0.0516		0.063	0.028	0.37
Water	0.219		0.100	0.273	
Water	0.119				
Blood	0.95				
Blood	0.52				
Blood	0.73				
Soil		0.32	0.0002	0.0004	
Soil		0.95	0.0003	0.0007	
Soil		1.0	0.0001	0.0001	
Alloy		0.074			
Alloy		0.12			
Synthetic mixture		0.13			
Synthetic mixture		0.52			
Synthetic mixture		0.72			
Food			0.79		
Food			0.68		
Urine				1.02	
Urine				0.84	
Tea					0.98
Wheat					0.76

a Tabulated F-value for (5, 5) degrees of freedom at *P*(0.98) is 5.72. *s*_1_ = standard deviation of proposed method, *s*_2_ = standard deviation of reference method,^15^*s*_3_ = standard deviation of reference method,^26^*s*_4_ = standard deviation of reference method,^31^*s*_5_ = standard deviation of reference method,^33^*s*_6_ = standard deviation of reference method.^28^

### Determination of manganese in synthetic mixtures

Several synthetic mixtures of varying compositions containing manganese(vii) and diverse ions of known concentrations were determined by the present method using EDTA as masking agent. The results were found to be highly reproducible. Accurate recoveries were achieved in all solutions in the range 98 ± 0.5 to 100 ± 0.0%. The reliability of our manganese–PTQA procedure was approved by quantitative recovery of manganese(vii) spiked in several synthetic mixtures containing manganese(vii) and diverse ions. The method has high precision and accuracy (*s* = ±0.01 for 0.5 μg L^−1^).

### Determination of manganese in certified reference materials

A 0.1 g amount of an alloy or steel sample containing 0.472–1.36% of manganese was weighed accurately and placed in a 50 mL Erlenmeyer flask in presence of excess oxidizing agent following a method recommended by Parker.^48^ To it, 10 mL of 20% (w/v) sulfuric acid was added and the mixture was heated gently. The residual carbides were decomposed by gently simmering with 1 mL of concentrated H_3_PO_4_ and 10 mL of concentrated HNO_3_. Then a further 2 mL of 1 + 1 H_2_SO_4_ and 2 mL 2% w/v freshly prepared persulphate were added and the solution was evaporated carefully to dense white fumes of sulphur trioxide, then cooled to room temperature (25 ± 5 °C). After suitable dilution with de-ionized water, the contents of the Erlenmeyer flask were warmed so as to dilute the soluble salts. The solution was then cooled and neutralized with dilute NH_4_OH in presence of 1–2 mL of 0.01% (w/v) EDTA solution. The resulting solution was filtered if necessary, through a Whatman no. 40 filter paper into a 100 mL calibrated flask. The residue (silica tungstanic acid) was washed with a small volume of hot 1 + 99 H_2_SO_4_, followed by water; the volume was made up to mark with de-ionized water.

A suitable aliquot (1–2 mL) of the above-mentioned solution was taken into a 10 mL calibrated flask and the manganese(vii) content was determined; as described under procedure using tartrate or EDTA as masking agent. The proposed procedure for the spectrofluorimetric determination of manganese was applied to the analysis of estuarine sediment (NIST-SRM-1646) and human hair (CRM-BCR-397) CRMs obtained from the National Resource Council of Canada using tartrate or EDTA as a masking agent, following a method recommended by Sun *et al.*^49^ Based on five replicate analyses, average manganese concentration determined by spectrofluorimetric method was in excellent agreement with the certified values. The results are given in Table 3.

### Determination of manganese in environmental water samples

Each filtered (with Whatman no. 40) environmental sample (25 mL) contained in a 50 mL Pyrex beaker were added 1 mL of concentrated H_2_SO_4_, 1 mL of concentrated H_3_PO_4_ and 2 mL of concentrated HNO_3_ and the mixture was heated on a hot plate until white fumes of sulphur trioxide; in the presence of freshly prepared 2 mL of 2% (w/v) persulphate solution in a fume cupboard to oxidize manganese(ii) to manganese(vii) following a method recommended by Greenberg *et al.*^50^

An aliquot (1–2 mL) of this water sample was pipetted into a 10 mL calibrated flask and the manganese content was determined as described under the general procedure using tartrate or EDTA as masking agent. The results of analyses of environmental water samples from various sources for manganese are shown in Table 4.

Spectrofluorimetric methods for determination of manganese in natural and sea-water does not require any preconcentration of manganese.^51^ The concentration of manganese in natural and sea water is a few μg L^−1^ in developed countries.^52^ The mean concentration of manganese found in U.S. drinking water is greater than 20 μg L^−1^.^52^

### Determination of manganese in biological samples

Human blood (1–2 mL) or urine (5–10 mL) or hair (1–2 g) sample was taken into a 100 mL micro-Kjeldahl flask. A glass bead and 10 mL of concentrated nitric acid were added, and the flask was placed on the digester under gentle heating. The sample was digested in the presence of an excess persulphate solution according to the method recommended by Stahr.^53^ As the heating process continued 1 mL of H_2_SO_4_ is added and heated for about 0.5 hour to dense white fumes of sulphur trioxide.

When the initial brisk reaction was completed, the solution was removed and cooled at room temperature. A 1 mL volume of 3.33 M H_3_PO_4_ was carefully added, followed by the addition of 2 mL of 2% (w/v) freshly prepared ammonium persulphate solution to oxidize Mn(ii) to Mn(vii) and heating was continued to dense white fumes, while repeating the nitric acid addition, if necessary. The solution was then cooled and neutralized with a dilute NH_4_OH. The final solution is made up to the mark with de ionized water.

A suitable aliquot (1–2 mL) of the final solution was pipetted out into a 10 mL calibrated flask and the manganese content was determined as described under procedure using EDTA or tartrate as masking agent. The results of biological analyses by the spectrofluorimetric method were found to be in excellent agreement with those obtained by AAS. The results are shown in Table 5.

The abnormally high value for the manganism patient is probably due to the involvement of high manganese concentrations with As and Zn. The occurrence of such high manganese contents are also reported in manganism patient from some developed countries.^54^

### Determination of manganese in soil samples

An air-dried homogenized soil sample (10 g) was accurately weighed and placed in a 100 mL micro-Kjeldahl flask. The sample was digested in the presence of an oxidizing agent (2 mL of 2% freshly prepared ammonium persulphate solution) following method recommended by Jackson.^55^ The solution was cooled and in the resulting solution 1 mL of 3.33 M H_3_PO_4_, 2 mL of 2% (w/v) freshly prepared ammonium persulphate was added. The solution was heated for 5–10 min to affect complete oxidation from manganese(ii) to manganese(vii). The solution is reduced in volume by evaporation to eliminate excess persulphate.

The content of the flask was filtered through a Whatman no. 40 filter paper into 25 mL calibrated flask and neutralized with dilute NH_4_OH solution in the presence of 1–2 mL of 0.01% (w/v) EDTA solution. The resulting solution was then diluted up to the mark with de-ionized water.

A suitable aliquot (1 mL) of the final solution was pipetted out into a 10 mL calibrated flask and the manganese content was determined as described under procedure using tartrate or EDTA as masking agent. The manganese content was then determined by the above procedure and quantified from a calibration graph prepared concurrently. The results are shown in Table 6. The average value of manganese in Bangladesh surface soil was found to be 43.29 mg kg^−1^.^56^

### Determination of manganese in pharmaceutical samples

Finished pharmaceutical samples (each Mn containing 1 mg tablet or 5 mL syrup or required weight) were quantitatively taken in a 50 mL beaker and digested in the presence of excess oxidizing agent following a method recommended by Ahmed *et al.*^57^ 10 mL of concentrated nitric acid is added and heated to dryness and then added 10 mL of 20% (v/v) of H_2_SO_4_ and heated to white fume. In the resulting solution 1 mL of 3.33 M H_3_PO_4_, 2 mL of 2% (w/v) freshly prepared ammonium persulphate is added. The solution is heated for 5–10 min to effect complete oxidation of manganese(ii) to manganese(vii). The solution is reduced in volume by evaporation to eliminate excess persulphate. The solution was then cooled and neutralized with dilute NH_4_OH in the presence of 1–2 mL of 0.01% (w/v) EDTA solution. The resulting solution was then filtered and quantitatively transferred into a 25 mL calibrated flask and made up to the mark with de-ionized water.

An aliquot (1–2 mL) of this digested sample was pipetted into a 10 mL calibrated flask and then manganese content was determined as described under the general procedure using tartrate or EDTA as a masking agent. The results of some pharmaceutical analyses are in excellent agreement with the reported values. The analyses of pharmaceutical samples from several Pharmaceutical Companies for manganese are given in Table 7.

### Determination of manganese in vegetable and fruit samples

The vegetable and fruit samples collected prior to the determination were pretreated in the following way: edible portion of samples was first washed clean with tap water followed by rewashing with de-ionized water. After removing de-ionized water from the surface of vegetables and fruits, the samples were cut into small pieces and dried at 65 °C in oven. An air dried vegetables and fruits samples (10 gm) were taken in a 100 mL micro-Kjeldahl flask in presence of oxidizing agent and digested following a method recommended by Stahr^53^ and 10 mL of concentrated nitric acid were added and the flask was placed on the digester under gentle heating. When the initial brisk reaction was over, the solution was removed and cooled at room temperature. 1 mL volume of concentrated sulfuric acid was added carefully, followed by the addition of 2 mL of concentrated HF, and heating was continued for at least 1/2 h and then cooled. In the resulting solution 1 mL of 3.33 M H_3_PO_4_, 2 mL of 2% (w/v) freshly prepared ammonium persulphate is added. The mixture of each foodstuff was heated below the boiling point for 5–10 min to oxidize manganese(ii) to manganese(vii). The resulting solution was then cooled and neutralized with dilute NH_4_OH in presence of 1–2 mL of (w/v) EDTA solution. The resulting solution was filtered and quantitatively transferred into a 25 mL calibrated flask and mixed well and made up to the mark with de ionized water.

A suitable aliquot (1–2 mL) of the final solution was pipetted into a 10 mL calibrated flask and the manganese content was determined as described under the procedure using tartrate as masking agent. High value of manganese for *Allium cepa* (onion) is probably due to the involvement of high manganese concentration in the soil.^58^ The results of vegetable and fruit analysis by spectrofluorimetric method were also found to be in excellent agreement with those obtained by AAS. The results are shown in Table 8.

### Determination of manganese in food and tea samples

The food samples used were rice, wheat and tea and these were used under dry conditions. Each sample was first ground in a mortar. Tea samples (0.1 g) or rice and wheat samples (1.0 g) were weighed accurately and placed in a porcelain crucible and charred in an electric furnace; the sample was ashed at 555 °C in a muffle furnace in presence of excess oxidizing agent following a method recommended by Stahr.^53^ 2 mL of HCl and 10 mL of water were added to the ash. The mixture of each foodstuff was heated below the boiling point for a moment. In the resulting solution 1 mL of 3.33 M H_3_PO_4_, 2 mL of 2% (w/v) freshly prepared ammonium persulphate is added. The solution is heated for 5–10 min to effect complete oxidation from Mn(ii) to Mn(vii). The solution was then cooled and neutralized with dilute NH_4_OH in presence of 1–2 mL of 0.01% (w/v) EDTA solution and filtered. The resulting solution was quantitatively transferred transferred into a 25 mL calibrated flask and mixed well and made up to the mark with de-ionized water.

A suitable aliquot (1–2 mL) of the final solution was pipetted out into a 10 mL calibrated flask and the manganese content was determined as described under procedure using tartrate as masking agent. The results of food analyses by the spectrofluorimetric method were also found to be in excellent agreement with those obtained by ICP-MS. The results are shown in Table 9.

High value of manganese for *Camellia sinensis* (green tea) is probably due to the involvement of high manganese concentration in the soil.

### Determination of manganese(ii) and manganese(vii) speciation in mixtures

Suitable aliquots (1–2 mL) of manganese (VII + II) mixtures (preferably 1 : 1, 1 : 5, 1 : 10) were taken in a 25 mL Pyrex beaker. In this solution 1 mL of 3.33 M H_3_PO_4_, 1 mL of 10^−4^ M silver nitrate and 2 mL of 2% (w/v) freshly prepared ammonium persulphate is added. The solution (4–5 mL) is heated for 5–10 min to effect complete oxidation to convert all Mn(ii) into Mn(vii). The solution is reduced in volume by evaporation to half of its initial volume to eliminate excess persulphate. The solution was then cooled and neutralized with dilute NH_4_OH. The solution is taken quantitatively in to 10 mL volumetric flux and 1 mL of 1 × 10^−4^ M PTQA reagent solution was added followed by the addition of 1 mL of 0.025 M H_2_SO_4_ and 2 mL ethanol. It was made up to the mark with de-ionized water. The fluorescence intensity was measured after 5 min at 373 nm when excited at 319 nm, against a reagent blank. The total manganese content was calculated with the help of a calibration graph prepared concurrently.^58,59^

An equal aliquot (1–2 mL) of the above manganese (VII + II) mixture was taken into a 25 mL beaker. Neutralize the solution with dilute NH_4_OH in presence of 1–2 mL of 0.01% (w/v) tartrate solution. After, the content of the beaker was transferred quantitatively into a 10 mL volumetric flask, 1 mL of 1 × 10^−4^ M PTQA reagent solution was added, followed by the addition of 1 mL of 0.025 M H_2_SO_4_ and 2 mL ethanol. It was made up to the mark with de-ionized water. After 5 min the fluorescence intensity was measured at 373 nm when excited at 319 nm against a reagent blank, as before. The manganese concentration was calculated in μg L^−1^ or ng L^−1^ with the aid of a calibration graph. This gives a measure of manganese(vii) originally present in the mixture. This value was subtracted from that of the total manganese to get the manganese(ii) present in the mixture. The results were found to be highly reproducible. The occurrence of such reproducible results is also reported for different oxidation states of manganese.^60^ The results of a set of determination are given in Table 10.

The present method was compared with some reported methods^15,26,28,31,33^ statistically. It was found that present method is much superior those of the reported methods. The results are shown in Table 11.

## Conclusions

A new simple, ultra sensitive, highly selective and inexpensive spectrofluorimetric method with the manganese(vii)–PTQA system was developed for the determination of manganese in some real, environmental, biological, soil, food, vegetable, fruit and pharmaceutical samples, for continuous monitoring to establish the nano-trace levels of manganese in different samples matrices. Compared with other methods^4–40^ in the literature, the proposed method has several remarkable analytical characteristics. Firstly, the proposed method is highly sensitive that amount of ng L^−1^ of manganese can be determined without preconcentration. Secondly, the proposed method is very simple, rapid, and stable. The reaction of manganese(vii) with PTQA is completed rapidly in 1 min at room temperature so it does not involve any stringent reaction conditions and offer the advantages of stability of fluorescence intensity (24 h). Thirdly, the method has added the advantage of determining individual amounts of Mn(vii) and Mn(ii). With suitable masking agents, the reaction can be made highly selective. The proposed method using PTQA in aqueous solutions not only is one of the most sensitive methods for the determination of manganese but also is excellent in terms of selectivity and simplicity. Therefore, this method will be successfully applied to the monitoring of nano-trace amounts of manganese in real, environmental, biological, soil, fruit, vegetables, food and pharmaceutical samples.

## Conflicts of interest

There are no conflicts to declare.

## Supplementary Material
